# The Safest Hypnotic for Children

**Published:** 1898-10

**Authors:** 


The Safest Hypnotic for Children.—Although sleeplessness in children can frequently be relieved by non-medicinal measures, it not rarely happens that the use of hypnotics can not be avoided, and under these circumstances the question will present itself to the physicians as to what remedy is the safest and most efficient for these little patients. It is important, of course, to select a hypnotic which will be entirely devoid of narcotic influences and which will not disturb the digestion, weaken the nervous system, or leave behind a train of unpleasant after-effects. In reviewing the list of the older as well as more recent hypnotics, trional suggests itself as the one best adapted for the various forms of insomnia in childhood. Dr. J. McGee, of Cleveland, Ohio, in a recent study of the action of drugs in children (*American Therapist*) expresses himself as follows in regard to this remedy: "It does not irritate the kidneys, and is very efficient when the insomnia is nervous in its origin. The dose at one year is from two to five grains, and this is gradually increased with each year. Twenty grains have been safely given to a child of ten or twelve years. This really represents an average adult dose, but it shows the tolerance and comparative safety of the remedy. It should be given shortly before retiring, as its effect is quite rapid, although the sleep is not so prolonged as that produced by sulfonal, which it equals in power, with the advantage of prompter action and freer from unpleasant after-effects."

